# Supplementary material for: First Evidence of Function for Schistosoma japonicum riok-1 and RIOK-1
Source: Pathogens. 2021 Jul 8;10(7):862. doi: 10.3390/pathogens10070862 (PMC8308690; doi:10.3390/pathogens10070862)
Supplement: Supplementary file 1 [file pathogens-10-00862-s001.zip › pathogens-1278202-supplementary.pdf]

Table S1. DNA sequences of oligonucleotide primers used in the present study. These primers were employed for the isolation of 3′cDNA of *Sj-riok-1* and for the amplification of DNA template for dsRNA synthesis of *Sj-riok-1*, non-specific *egfp* genes using PCR-based approaches and for real-time (RT) PCR quantification.

Name	Primer Sequence (5′-3′)
Riok1-F	TGAAGAACGAGAAGCAGATGA
Riok1-R	GCTGGTAGACACGGAGGATT
Riok1-ORF-F	ATGAAGAACGAGAAGCAGAT
Riok1-ORF-R	GGTGTCATTACTTTTTTATTTTAC
Riok1-qPCR-F	GTTTCTCAGCCTGTTCTTGC
Riok1-qPCR-R	GCTGGTGATACTCCCTTTTT
Egfp-qpCR-F	ATGGTGAGCAAGGGCGAGGA
Egfp-qpCR-R	GTGGTTGTCGGGCAGCAGCA
β-Tubulin-qPCR-F	GCGGGACAGTGTGGTAATCA
β-Tubulin-qPCR-R	ATGCGTTCAAGTTGTAAATCAGAG
dsRNA-riok1-F	<u>TAATACGACTCACTATAGGG</u>TGAAGAACGAGAAGCAGATGA
dsRNA-riok1-R	<u>TAATACGACTCACTATAGGGG</u>CTGGTAGACACGGAGGATT
dsRNA-egfp-F	<u>TAATACGACTCACTATAGGG</u>ATGGTGAGCAAGGGCGAGGA
dsRNA-egfp-R	<u>TAATACGACTCACTATAGGGG</u>TGGTTGTCGGGCAGCAGC

[a] underscore represents T7 promotor site.

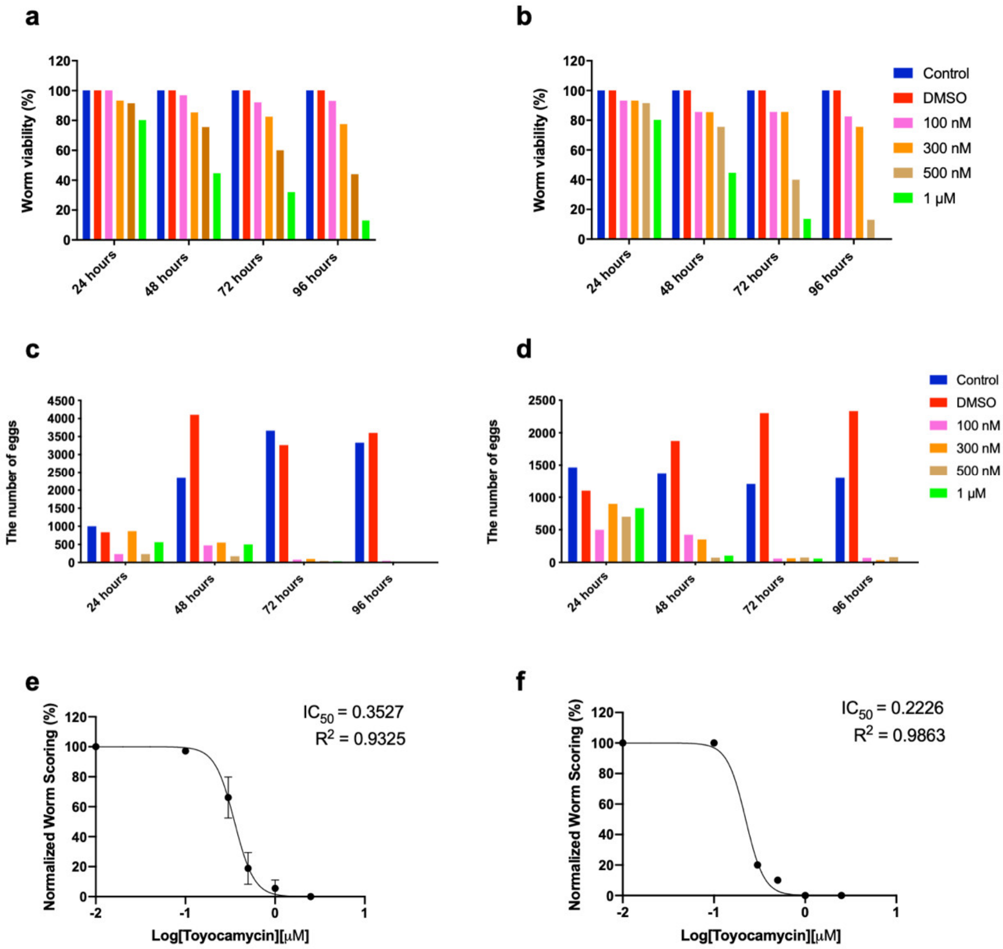

Figure S1. Preliminary data of toyocamycin on viability and egg production of 35-do and 28-do adult worms of *Schistosoma japonicum*. (**a**,**c**) Effect of toyocamycin treatment between 24 h and 96 h on viability and egg production of 35-do worms. (**b**,**d**) Effect of toyocamycin treatment between 24 h and 96 h on viability and egg production of 28-do worms. (**e**,**f**) IC50 value for worm viability scoring in response to toyocamycin at 96 h of 35-do and 28-do adult worms, respectively. Data without error bars represents *n* = 1.
